# Distribution of Tri-Ponderal Mass Index and its Relation to Body Mass Index in Children and Adolescents Aged 10 to 20 Years

**DOI:** 10.1210/clinem/dgaa030

**Published:** 2020-01-29

**Authors:** Hong Kyu Park, Young Suk Shim

**Affiliations:** 1 Department of Pediatrics, Gyeongsang National University Changwon Hospital, Changwon, Korea; 2 Department of Pediatrics, Hallym University Dongtan Sacred Heart Hospital, Hwaseong, Korea

**Keywords:** Tri-ponderal mass index, body mass index, obesity, cardiometabolic risk

## Abstract

**Context:**

Body mass index percentiles are widely used to determine overweight and obesity status in children and adolescents. Their limitations in clinical settings can be addressed.

**Objective:**

Reference ranges for the tri-ponderal mass index percentiles of Korean children and adolescents are presented for a comparison of their clinical variables with those of body mass index.

**Design:**

Cross-sectional study.

**Setting:**

Korea National Health and Nutrition Examination Survey, 2007–2016.

**Patients:**

Korean children and adolescents aged 10 to 20 years.

**Main Outcome Measures:**

The age- and sex-specific least mean square parameters (skewness, median, and coefficient of variation) for the tri-ponderal mass index of 9749 subjects aged 10 to 20 years.

**Results:**

The factors associated with metabolic syndrome, except diastolic blood pressure, were more likely to be worse in the subjects with tri-ponderal mass index values indicative of overweight status than in those with normal tri-ponderal mass index values. Body mass index tends to underestimate obesity-related comorbidities more than tri-ponderal mass index does.

**Conclusion:**

The tri-ponderal mass index standard deviation score may be advantageous when defining overweight and obesity in children and adolescents.

Overweight and obesity are states caused by the excessive accumulation of body fat. They are associated with various comorbidities, such as diabetes mellitus, cardiovascular diseases, and sleep apnea (1–3). The prevalence of obesity has been increasing worldwide in recent decades (4–6). Body fat can be measured directly using various imaging techniques, such as magnetic resonance imaging, computed tomography, and dual-energy X-ray absorptiometry (DXA); however, although these methods are accurate, they are not suitable for every individual because not only are they expensive and time-consuming, but the latter 2 carry a risk of radiation exposure. In addition, there are several indirect methods for measuring adiposity. Body mass index (BMI), waist circumference (WC), waist-to-hip ratio, and skin fold thickness are used as surrogate measures of adiposity. Among these measurements, BMI is well established for the purpose of screening for obesity in clinical practice. It was developed more than 100 years ago and it is calculated as the weight in kilograms divided by the height in meters squared. Practical guidelines usually classify obesity in terms of BMI. For adults, a BMI between 25.0 and 29.9 kg/m^2^ is defined as overweight and a BMI of 30 kg/m^2^ or higher is defined as obese (2). In children and adolescents, overweight and obesity are also evaluated and screened using BMI (7–9). The recently published guidelines for pediatric obesity recommend using normative BMI percentiles to diagnose overweight or obesity (10). A child or adolescent older than 2 years of age with a BMI greater than the 95th percentile for age and sex is considered obese and overweight is defined as a BMI between the 85th and 95th percentiles.

The ratio between the weight and height squared is relatively stable in adulthood; however, this ratio varies during childhood and adolescence because of differences in the rates of change in weight and height (11). The scaling power of height increases gradually up to approximately 3 during puberty and changes variously according to pubertal status. Although the BMI percentiles were implemented to resolve the disproportion of weight to squared height, because of the fundamental limitations of the original value, its ability to reflect body proportions seems to be limited. Because volume is proportional to the cube of length, the tri-ponderal mass index (TMI), which is calculated as the weight in kilograms divided by the height in meters cubed, can be considered. A recent study demonstrated that the TMI estimates body fat levels more accurately than BMI does in children and adolescents aged 8 to 17 years (12). The authors suggested a single TMI value as a cutoff for defining overweight.

This study aimed to determine the TMI values of Korean children and adolescents aged 10 to 20 years using nationally representative data. The clinical significance of TMI can be determined by comparing its variables related to metabolic syndrome with those of BMI.

## Materials and Methods

Data from the 2007–2016 Korea National Health and Nutrition Examination Survey (KNHANES) were analyzed in this study. The survey has been conducted by the Division of Chronic Disease Surveillance, Korean Centers for Disease Control and Prevention on a 3-year cycle since 1998 to assess the health and nutritional status of the noninstitutionalized civilian population of Korea (13). The KNHANES is a cross-sectional and nationally representative survey with a multistage and stratified probability sampling design. To enhance the statistical power of this analysis, data acquired from the full fourth (2007-2009), fifth (2010-2012), and sixth (2013-2015) cycles and the first year of the seventh cycle (2017) were combined. A total of 81 503 individuals were included. Of these subjects, 10 510 participants aged 10 to 20 years were included in the preliminary analysis. All subjects and their parents were interviewed at home after providing informed consent and underwent various examinations, including blood sampling. Those with incomplete records regarding the physical examination, including anthropometric measurements, blood pressure, and laboratory tests, including the lipid profile, were excluded. Those who were currently taking blood cholesterol-lowering medications were also excluded (n = 745). Blood samples were obtained by venipuncture after overnight fasting. The collected samples were immediately processed and refrigerated and were transported daily to the central laboratory (NEODIN Medical Institute, Korea) for analysis within 24 hours. From 2007 to 2008, serum total cholesterol (TC), high-density lipoprotein cholesterol (HDL-C), and triglyceride (TG) concentrations were measured enzymatically using ADIVIA1650 (Siemens, USA). Since 2009, TC, HDL-C, and TG have been enzymatically measured using a Hitachi Automatic Analyzer 7600 (Hitachi, Japan) with reagents (SEKISUI, Japan) by NEODIN Medical Institute. Low-density lipoprotein cholesterol (LDL-C) was calculated with the Friedewald equation (LDL-C = TC – [HDL-C + (TG ÷ 5)]) (14). For accuracy, samples with TG levels >400 mg/dL were also excluded. Thus, the final analytical sample consisted of 9749 subjects (5017 boys and 4732 girls). The database is available to the public at the KNHANES website (http://knhanes.cdc.go.kr). The study protocols of the 2007–2016 KNHANES were approved by the institutional review boards of the Korean Centers for Disease Control and Prevention. Informed consent was provided by all KNHANES subjects. This study was also approved by the institutional review board of Hallym University Dongtan Sacred Heart Hospital (number: 2019-08-015).

Anthropometric assessments, including height, weight, WC, and systolic and diastolic blood pressure (SBP and DBP, respectively) were performed by a trained expert. Height was measured to the nearest 0.1 cm using an electronic stadiometer (SECA, Germany). Weight was measured to the nearest 0.1 kg with an electronic scale (G-TECH, Korea). WC was measured to the nearest 0.1 cm using a calibrated measuring tape (SECA). SBP and DBP were measured 3 times to the nearest 1 mm Hg using a standard mercury sphygmomanometer. The SD scores (SDS) for height, weight, WC, and BMI were calculated using age- and sex-specific least mean square (LMS) parameters based on the 2017 growth reference values for Korean children and adolescents developed by the Korean Pediatric Society and the Korea Centers for Disease Control and Prevention (15). The subjects were categorized into 4 groups according to BMI: underweight (BMI was <3rd percentile), normal weight (BMI was ≥3rd percentile and <85th percentile), overweight (BMI was ≥85th percentile and <95th percentile), and obesity (BMI was ≥95th percentile). To investigate the relationship between obesity groups, the TMI reference values calculated in this study were used to also categorize the subjects into 4 groups as follows: underweight (TMI was <3rd percentile), normal weight (TMI was ≥3rd percentile and <85th percentile), overweight (TMI was ≥85th percentile and <95th percentile), and obesity (TMI was ≥95th percentile).

Lifestyle-related behaviors, such as alcohol consumption, smoking, household income, and residence, were assessed by means of a questionnaire. Information about alcohol consumption (drinkers vs. nondrinkers) and smoking status (smokers vs. nonsmokers) was collected with a self-administered questionnaire from subjects aged 12 years and older. Questionnaires on household income and area of residence (urban vs. rural) were administered by trained interviewers.

R, version 3.5.1 (The R Foundation for Statistical Computing, Austria), was used for statistical analysis. Continuous variables are expressed as mean and SD. The 3 LMS parameters (lambda for the Box-Cox power for skewness, mu for the median, and sigma for the generalized coefficient of variation) for TMI were estimated using the GAMLSS package (16). Age- and sex-specific parameters were smoothed with the LMS method using penalized likelihood in nonlinear regression to fit the curves as cubic splines, as proposed by Cole and Green (17). In addition, the age- and sex-specific values for the 3rd, 5th, 10th, 15th, 25th, 50th, 75th, 85th, 90th, 95th, and 97th percentiles are presented to provide a tool for assessing overweight and obesity in Korean children and adolescents. A 2-tailed *t*-test was performed to identify differences in clinical parameters between the BMI- and TMI-based categorization of overweight and obesity, whereas categorical variables were compared using the χ ^2^ test. Probability values less than .05 were considered statistically significant.

## Results

**Figure 1.**
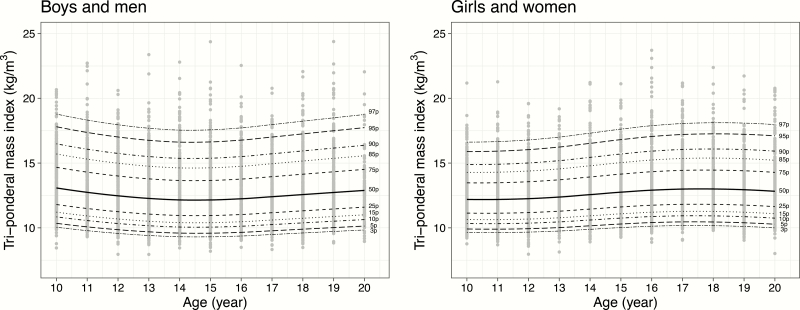
Tri-ponderal mass index for age percentiles for Korean children and adolescents aged 10 to 20 years.

Table 1 presents the clinical characteristics of the study population. The SDS of height and weight were significantly higher for boys than for girls. SBP, DBP, and serum glucose concentrations were higher for boys, and concentrations of TC, HDL-C, and LDL-C were higher for girls. Age- and sex-specific percentiles and the corresponding LMS variables for TMI are shown in Table 2 and Figure 1. The values of each percentile were relatively constant, regardless of age. The median estimates ranged from 12.149 to 13.052 kg/m^3^ for boys and men and 12.161 to 13.009 kg/m^3^ for girls and women. The third percentile values for TMI ranged from 9.307 to 10.025 kg/m^3^ for boys and 9.613 to 10.189 kg/m^3^ for girls. The 85th percentile values ranged from 14.621 to 15.671 kg/m^3^ for boys and 14.263 to 15.383 kg/m^3^ for girls. The 95th percentile values ranged from 15.361 to 16.453 kg/m^3^ for boys and 14.874 to 16.084 kg/m^3^ for girls. Table 3 presents the differences in the prevalences of underweight, normal weight, overweight, and obesity according to TMI- and BMI-based classifications. Notably, 53.2% of the 996 subjects who were categorized as overweight according to BMI were classified as normal weight by TMI. Boys tended to be misclassified more frequently than girls (61.0% vs. 43.6%; *P *< .01, χ ^2^, data not shown). Among the 1037 participants with obesity diagnosed on the basis of BMI, 49.3% were classified as overweight and 2.6% were categorized as normal weight when TMI was applied. The misclassification rates were also significantly higher in the male subjects than in the female subjects (54.2%, 3.8% vs. 42.4%, 0.9%; *P *< .01, χ ^2^, data not shown).

**Table 1.** Clinical Characteristics of the Study Population According to Sex (n = 9749)

	Boys and Men	Girls and Women	
	(n = 5017)	(n = 4732)	*P*
Age (y)	14.2 ± 2.9	14.5 ± 3.1	<.001
Height SDS	0.51 ± 1.08	0.36 ± 1.08	<.001
Weight SDS	0.28 ± 1.23	0.16 ± 1.14	<.001
WC SDS	−0.17 ± 1.24	−0.17 ± 1.12	.779
BMI SDS	0.05 ± 1.31	−0.01 ± 1.19	.012
TMI SDS	0.00 ± 1.00	0.00 ± 1.00	.870
SBP (mm Hg)	109.1 ± 10.7	104.1 ± 9.3	<.001
DBP (mm Hg)	66.9 ± 9.7	65.7 ± 8.4	<.001
Glucose (mg/dL)	90.39 ± 7.5	88.88 ± 8.9	<.001
TC (mg/dL)	156.1 ± 27.0	163.8 ± 26.5	<.001
HDL-C (mg/dL)	49.8 ± 9.8	52.7 ± 10.1	<.001
TG (mg/dL)	84.5 ± 44.9	85.0 ± 43.5	.666
LDL-C (mg/dL)	89.4 ± 23.2	94.1 ± 23.0	<.001
Alcohol consumption (%)	509 (10.2)	446 (9.4)	.245
Smoking (%)	694 (13.8)	260 (5.5)	<.001
Household income (<2nd quartile)	558 (11.7%)	569 (12.0%)	.666
Rural residence (%)	822 (16.4)	741 (15.7)	.343

BMI, body mass index; DBP, diastolic blood pressure; HDL-C, high-density lipoprotein cholesterol; LDL-C, low-density lipoprotein cholesterol; SBP, systolic blood pressure; SDS, SD score; TC, total cholesterol; TG, triglyceride; TMI, tri-ponderal mass index; WC, waist circumference.

**Table 2.** LMS Values and Specific Percentile Limits for TMI According to Age for Boys and Girls

TMI (kg/m^3^)														
				Percentile										
Age	n	L	S	3rd	5th	10th	15th	25th	50th (M)	75th	85th	90th	95th	97th
Boys and men														
10	574	-1.021	0.161	10.025	10.325	10.824	11.188	11.775	13.052	14.643	15.671	16.453	17.771	18.747
11	590	-1.021	0.161	9.817	10.111	10.600	10.958	11.534	12.788	14.353	15.363	16.134	17.430	18.392
12	574	-1.021	0.162	9.581	9.868	10.347	10.697	11.261	12.489	14.022	15.012	15.768	17.040	17.984
13	549	-1.021	0.162	9.392	9.674	10.145	10.489	11.043	12.251	13.759	14.735	15.479	16.733	17.663
14	547	-1.021	0.163	9.307	9.588	10.055	10.398	10.948	12.149	13.650	14.621	15.361	16.611	17.538
15	488	-1.021	0.163	9.307	9.588	10.057	10.400	10.952	12.156	13.663	14.638	15.382	16.638	17.571
16	436	-1.021	0.164	9.365	9.649	10.121	10.467	11.024	12.240	13.762	14.748	15.500	16.771	17.715
17	429	-1.021	0.164	9.474	9.762	10.241	10.592	11.157	12.391	13.936	14.938	15.703	16.995	17.956
18	390	-1.021	0.165	9.625	9.918	10.405	10.763	11.339	12.597	14.173	15.194	15.976	17.295	18.277
19	302	-1.021	0.165	9.757	10.055	10.551	10.914	11.500	12.779	14.383	15.423	16.219	17.564	18.565
20	138	-1.021	0.166	9.812	10.112	10.612	10.978	11.569	12.859	14.478	15.530	16.334	17.694	18.706
Girls and women														
10	510	-1.045	0.141	9.669	9.928	10.354	10.664	11.157	12.214	13.498	14.307	14.915	15.919	16.649
11	502	-1.045	0.142	9.613	9.871	10.298	10.608	11.102	12.161	13.449	14.263	14.874	15.885	16.621
12	486	-1.045	0.143	9.645	9.906	10.336	10.649	11.149	12.220	13.525	14.350	14.971	15.998	16.746
13	482	-1.045	0.144	9.759	10.024	10.463	10.782	11.291	12.384	13.717	14.561	15.196	16.249	17.017
14	484	-1.045	0.145	9.888	10.159	10.606	10.931	11.451	12.567	13.931	14.796	15.447	16.527	17.316
15	408	-1.045	0.146	10.014	10.289	10.745	11.076	11.606	12.745	14.140	15.025	15.693	16.801	17.611
16	427	-1.045	0.147	10.131	10.411	10.875	11.213	11.752	12.915	14.339	15.245	15.928	17.064	17.895
17	418	-1.045	0.148	10.189	10.473	10.942	11.284	11.831	13.009	14.456	15.377	16.072	17.229	18.077
18	333	-1.045	0.149	10.164	10.448	10.919	11.263	11.812	12.997	14.454	15.383	16.084	17.254	18.111
19	360	-1.045	0.150	10.086	10.370	10.840	11.183	11.732	12.918	14.377	15.309	16.014	17.189	18.052
20	322	-1.045	0.151	10.006	10.289	10.759	11.101	11.650	12.835	14.297	15.232	15.939	17.121	17.990

LMS, least mean square; TMI, tri-ponderal mass index.

**Table 3.** The Relationship Between TMI and BMI

	TMI				
BMI	Underweight	Normal Weight	Overweight	Obesity	Total
Boys and men					
Underweight (%)	105 (52.5)	95 (47.5)	0 (0)	0 (0)	200 (100)
Normal weight (%)	33 (0.9)	3612 (98.6)	20 (0.5)	0 (0)	3665 (100)
Overweight (%)	0 (0)	335 (61.0)	213 (38.8)	1 (0.2)	549 (100)
Obesity (%)	0 (0)	23 (3.8)	327 (54.2)	253 (42.0)	603 (100)
Total (%)	138 (2.8)	4065 (81.0)	560 (11.2)	254 (5.1)	5017 (100)
Girls and women					
Underweight (%)	112 (57.1)	84 (42.9)	0 (0)	0 (0)	196 (100)
Normal weight (%)	24 (0.7)	3586 (98.1)	45 (1.2)	0 (0)	3655 (100)
Overweight (%)	0 (0)	195 (43.6)	240 (53.7)	12 (2.7)	447 (100)
Obesity (%)	0 (0%)	4 (0.9)	184 (42.4)	244 (56.7)	434 (100)
Total (%)	136 (2.9)	3869 (81.8)	469 (9.9)	258 (5.5)	4732 (100)
All participants					
Underweight (%)	217 (54.8)	179 (45.2)	0 (0)	0 (0)	396 (100)
Normal weight (%)	57 (0.8)	7198 (98.3)	65 (0.9)	0 (0)	7320 (100)
Overweight (%)	0 (0)	530 (53.2)	453 (45.5)	13 (1.3)	996 (100)
Obesity (%)	0 (0)	28 (2.6)	511 (49.3)	499 (48.1)	1037 (100)
Total (%)	274 (2.8)	7934 (81.4)	1029 (10.6)	512 (5.3)	9749 (100)

Underweight, BMI or TMI <3rd percentile; normal weight, BMI or TMI ≥3rd percentile and <85th percentile; overweight, BMI or TMI ≥85th percentile and <95th percentile; obesity, BMI or TMI ≥95th percentile.

BMI, body mass index; TMI, tri-ponderal mass index.

The differences in clinical characteristics according to TMI classification within the same BMI group are shown in Tables 4 and 5. Although the SDS for height was significantly higher in the normal weight TMI group regardless of BMI categorization and sex, the SDS for weight was lower in the normal weight TMI group among the subjects with normal weight according to BMI and higher in the normal-weight TMI group among the subjects with overweight according to BMI. It may be assumed that TMI can discern individuals with tall stature from heavier individuals. Among the subjects with normal weight BMI values (Table 4), TC and TG concentrations were higher in the boys with overweight TMI values than in those with normal weight TMI values (174.4 mg/dL vs. 153.6 mg/dL and 101.9 mg/dL vs. 77.4 mg/dL, respectively). The girls with overweight TMI values had lower concentrations of HDL-C (50.1 mg/dL vs. 53.5 mg/dL) and higher concentrations of TG (102.8 mg/dL vs. 81.4 mg/dL) than the girls with normal weight TMI values. Among the subjects with overweight BMI values (Table 5), the concentrations of TC and LDL-C were significantly higher for the boys with overweight TMI values than for those with normal weight TMI values (169.8 mg/dL vs. 157.5 mg/dL and 101.7 mg/dL vs. 90.8 mg/dL, respectively). The DBP and HDL-C concentrations were lower for the girls with overweight TMI values than for those with normal weight TMI values (65.5 mm Hg vs. 68.1 mm Hg and 49.5 mg/dL vs. 51.9 mg/dL, respectively). The TG concentrations of the girls with overweight TMI values were higher than those of the girls with normal weight TMI values (96.5 mg/dL vs. 82.6 mg/dL).

**Table 4.** Differences in Clinical Characteristics According to the TMI Classification of the Study Population with Normal BMI (≥3rd and <85th percentile)

	Boys and Men			Girls and Women		
	Normal TMI (n = 3612)	Overweight TMI (n = 20)	*P*	Normal TMI (n = 3586)	Overweight TMI (n = 45)	*P*
Age (y)	14.2 ± 2.9	13.1 ± 2.3	.043	14.5 ± 3.1	12.8 ± 3.2	<.001
Height SDS	0.45 ± 1.04	-1.04 ± 1.01	<.001	0.34 ± 1.03	-1.07 ± 1.33	<.001
Weight SDS	-0.09 ± 0.82	0.18 ± 0.50	.027	-0.11 ± 0.80	0.17 ± 0.75	.020
WC SDS	-0.54 ± 1.86	0.54 ± 0.37	<.001	-0.43 ± 1.68	0.12 ± 1.73	.029
BMI SDS	-0.40 ± 0.75	0.89 ± 0.12	<.001	-0.34 ± 0.71	0.85 ± 0.21	<.001
TMI SDS	-0.30 ± 0.65	1.14 ± 0.09	<.001	-0.24 ± 0.65	1.16 ± 0.12	<.001
SBP (mm Hg)	107.8 ± 10.2	106.2 ± 8.9	.497	103.4 ± 8.9	104.1 ± 7.3	.527
DBP (mm Hg)	66.4 ± 9.5	64.3 ± 8.1	.327	65.4 ± 8.3	63.4 ± 7.4	.124
Glucose (mg/dL)	90.0 ± 6.9	91.2 ± 8.2	.485	88.4 ± 6.8	89.0 ± 8.1	.620
TC (mg/dL)	153.6 ± 25.5	174.4 ± 35.3	.002	163.0 ± 26.0	169.5 ± 30.9	.138
HDL-C (mg/dL)	51.0 ± 9.8	50.8 ± 11.5	.951	53.5 ± 10.1	50.1 ± 9.8	.045
LDL-C (mg/dL)	87.2 ± 21.6	103.2 ± 33.0	.092	93.2 ± 22.4	98.8 ± 25.4	.135
TG (mg/dL)	77.4 ± 41.4	101.9 ± 50.7	.028	81.4 ± 40.2	102.8 ± 56.2	.029

BMI, body mass index; DBP, diastolic blood pressure; HDL-C, high-density lipoprotein cholesterol; LDL-C, low-density lipoprotein cholesterol; SBP, systolic blood pressure; SDS, SD score; TC, total cholesterol; TG, triglyceride; TMI, tri-ponderal mass index; WC, waist circumference.

**Table 5.** Differences in Clinical Characteristics According to the TMI Classification of the Study Population with Overweight BMI (≥85th and <95th Percentile)

	Boys and Men			Girls and Women		
	Normal TMI (n = 335)	Overweight TMI (n = 213)	*P*	Normal TMI (n = 195)	Overweight TMI (n = 240)	*P*
Age (y)	13.8 ± 3.0	13.6 ± 2.5	.243	15.1 ± 3.1	14.1 ± 3.0	.001
Height SDS	1.19 ± 0.88	0.04 ± 0.87	<.001	1.20 ± 0.92	0.06 ± 0.91	<.001
Weight SDS	1.54 ± 0.43	1.12 ± 0.46	<.001	1.54 ± 0.41	1.17 ± 0.44	<.001
WC SDS	1.02 ± 0.67	0.88 ± 0.52	.008	1.03 ± 0.54	1.01 ± 1.01	.834
BMI SDS	1.26 ± 0.16	1.40 ± 0.16	<.001	1.22 ± 0.13	1.38 ± 0.17	<.001
TMI SDS	0.84 ± 0.15	1.20 ± 0.13	<.001	0.85 ± 0.15	1.25 ± 0.15	<.001
SBP (mm Hg)	112.4 ± 10.6	112.4 ± 9.8	.969	106.5 ± 8.6	105.2 ± 9.3	.137
DBP (mm Hg)	67.5 ± 9.8	67.8 ± 9.5	.700	68.1 ± 7.6	65.5 ± 8.1	.001
Glucose (mg/dL)	92.5 ± 12.4	91.0 ± 6.5	.152	90.5 ± 19.5	90.3 ± 6.7	.927
TC (mg/dL)	157.5 ± 27.5	169.8 ± 32.5	<.001	162.1 ± 27.3	166.4 ± 25.9	.112
HDL-C (mg/dL)	46.4 ± 8.7	47.3 ± 9.4	.274	51.9 ± 9.9	49.5 ± 8.9	.013
LDL-C (mg/dL)	90.8 ± 22.8	101.7 ± 30.6	<.001	93.8 ± 23.7	97.8 ± 23.1	.098
TG (mg/dL)	101.5 ± 57.5	105.1 ± 60.4	.511	82.6 ± 45.7	96.5 ± 47.5	.004

BMI, body mass index; DBP, diastolic blood pressure; HDL-C, high-density lipoprotein cholesterol; LDL-C, low-density lipoprotein cholesterol; SBP, systolic blood pressure; SDS, SD score; TC, total cholesterol; TG, triglyceride; TMI, tri-ponderal mass index; WC, waist circumference.

## Discussion

In children and adolescents, weight varies with sex and age, and height distribution also varies according to puberty stage within the same age (18). Therefore, BMI is not constant across this age group. There is an increase in BMI at approximately 6 years of age, which is referred to as adiposity rebound. Then, the BMI reference curve displays a steep rise during puberty, and the increase continues until adulthood. Hence, it is reasonable to define overweight and obesity using BMI percentiles in these age groups, although BMI itself is not intuitive or simple. Childhood obesity is known to be related to obesity in adulthood and various adult comorbidities (19–21). There is a report indicating that obesity during youth is known to be a predictor of obesity in adulthood, regardless of whether the parents are obese (22). Additionally, Freedman et al. (23) demonstrated that the magnitude of the association depends on the relative fatness of the child.

Although BMI has been used as a reasonable proxy for obesity because of its simplicity of calculation and its relation to adverse outcomes, the limitations of BMI seem to necessitate the consideration of a better surrogate. BMI does not distinguish fat from muscle, bone, and other lean body mass (24, 25). In the past, before the “obesity epidemic,” estimating body fat with BMI may have been more feasible because fatness was not profoundly common in the general population. However, recently, several studies have demonstrated a poor correlation of BMI with percent body fat in children and adolescents. A meta-analysis assessed the performance of BMI and revealed that BMI values used to diagnose obesity showed a sensitivity of 73% in children and adolescents, suggesting that more than one-quarter of children and adolescents with an excess body fat percentage were misclassified by BMI (26). Vanderwall et al. (27) also reported that BMI SDS showed a weak relationship with percent body fat in children and adolescents. Another meta-analysis that included studies performed in adults demonstrated a low sensitivity for the identification of adiposity using common BMI cutoff values (28). In that study, the BMI cutoff values did not identify one-half of the subjects with excess body fat. Yusuf et al. (29) reported that BMI underestimated the number of people for whom fatness affects health. More recently, Peterson et al. (12) compared BMI and TMI with regard to estimating body fat in adolescents. According to their study, TMI estimated percent body fat more accurately than BMI did in non-Hispanic white adolescents aged 8 to 17 years and was more sensitive than BMI SDS for the diagnosis of overweight in adolescents. Furthermore, TMI, unlike BMI, did not need to be calculated to yield the SDS. De Lorenzo et al. (30) evaluated TMI as a predictor of percent body fat in Italian children and adolescents. They also found that TMI was more closely correlated than BMI with percent body fat and presented a higher area under the receiver operating characteristic curve. Both studies evaluated body fat DXA as a reference measure for TMI, and DXA is widely accepted for assessing body composition. However, we could not use body composition as a standard for measuring the accuracy of BMI and TMI to assess body fat. Nevertheless, this study found that TMI was more beneficial in discriminating metabolic risk factors than BMI was. Considering that body fat is significantly associated with cardiometabolic risk (31, 32), this study supports previous findings that TMI estimated percent body fat more accurately than BMI did.

In this study, TMI values for Korean children and adolescents aged 10 to 20 years were determined using nationally representative data, and the LMS method was applied to generate sex-specific reference tables with age-specific percentiles. The clinical variables related to metabolic syndrome were compared according to classifications by BMI and TMI. Except for DBP, the factors associated with metabolic syndrome were more likely to be worse in the subjects who were overweight according to TMI values than in those with normal weight TMI values within the same BMI classification (Table 4, 5), which suggests that in adolescents, BMI tends to underestimate obesity-related comorbidities more than TMI does. This finding seems to be compatible with the low sensitivity of BMI observed in previous studies. Although the parameters for TMI estimated by LMS approximation were relatively constant regardless of age, the farther away from the median the estimate was, the greater the deviation of each percentile. Therefore, the SDS of TMI may be suitable for the diagnosis of overweight and obesity. Two previous reports also presented fairly stable TMI values over time in children and adolescents (12, 33). Although a previous study by Peterson et al. (12) suggested that a fixed cutoff value was more accurate than BMI SDS for the classification of overweight status, the study by Ashley-Martin et al. (33) demonstrated that the use of a single sex-specific TMI cutoff value for obesity was not appropriate because of the increased variability of the measure according to age. They reported that the 97th percentile TMI values of subjects aged 10 to 20 years ranged from approximately 18.5 to 20.5 kg/m^3^ for boys and men and 18.5 to 23.0 kg/m^3^ for girls and women.

Several limitations need to be considered. Although the data used in this analysis were nationally representative, the BMI SDS was calculated using a growth reference for Korean children and adolescents that was based on measurements collected from different populations in 1997 and 2005. Therefore, the mean and SDS of height and weight in this study population were not exactly 0 and 1, respectively. It may be assumed that there have been increases in the height and weight of the population. Because growth reference charts are developed with a focus on showing how children should grow, they may not reflect the current status (15). This may need to be considered when interpreting the results of this study. In generating reference curves using cross-sectional data, a secular trend may be considered because it is related to reliability. The global prevalence of childhood obesity has been increasing in the past few decades (34). Given this circumstance, the reference curve of TMI will shift upward. Accordingly, the clinical implication of the reference curve will be affected. Although the prevalence of childhood obesity in Korea has been sustained since the early 2000s, the prevalence of extreme obesity has been increasing (35, 36). Combining data may have had an effect on the skewness of the curve in this study. Additionally, changes in adiposity according to pubertal stage could not be traced because the national survey does not collect data on pubertal milestones. Moreover, this was a cross-sectional study, and the data do not contain longitudinal information. Considering these characteristics, stable TMI values within a narrow range may be a good parameter on which to base the diagnosis of overweight and obesity.

The term obesity refers to adiposity. Body weight is not correlated with adiposity, and BMI tends to place more emphasis on body weight than TMI does. Because the cutoff values for overweight and obesity vary considerably with age in both sexes, the TMI SDS, rather than a fixed value, will be expected to have better performance for predicting obesity-related comorbidities in later adulthood. Therefore, it could be appropriate to use TMI SDS to define overweight and obesity in children and adolescents. Studies on optimal ranges of TMI SDS for measuring obesity-related adverse events in later life can improve long-term health outcomes.

## Abbreviations

BMI: body mass index
DBP: diastolic blood pressure
DXA: dual-energy X-ray absorptiometry
HDL-C: high-density lipoprotein cholesterol
KNHANES: Korea National Health and Nutrition Examination Survey
LDL-C: low-density lipoprotein cholesterol
LMS: least mean square
SBP: systolic blood pressure
SD: SD score
TC: total cholesterol
TG: triglyceride
TMI: tri-ponderal mass index
WC: waist circumference

## Additional Information

***Disclosure Summary:*** The authors have no financial relationships relevant to this article.

***Data Availability:*** All data generated or analyzed during this study are included in this published article or in the data repositories listed in References.
